# A Tumor Microenvironment Responsive Nanotheranostics Agent for Magnetic Resonance Imaging and Synergistic Photodynamic Therapy/Photothermal Therapy of Liver Cancer

**DOI:** 10.3389/fchem.2021.650899

**Published:** 2021-04-07

**Authors:** Yuwan Zhu, Mo Deng, Nannan Xu, Yingjun Xie, Xuewen Zhang

**Affiliations:** ^1^ Department of Hepatobiliary and Pancreatic Surgery, The Second Hospital of Jilin University, Changchun, Jilin, China; ^2^ State Key Laboratory of Rare Earth Resource Utilization, Changchun Institute of Applied Chemistry (CIAC), Chinese Academy of Sciences (CAS), Changchun, China; ^3^ Department of Clinical Laboratory, The Second Hospital of Jilin University, Changchun, China; ^4^ Department of Radiology, The Second Hospital of Jilin University, Changchun, China

**Keywords:** reactive oxygen species, nanomaterials, photodynamic therapy, photothermal therapy, magnetic resonance imaging, manganese oxide

## Abstract

Surgery is the main treatment for liver cancer in clinic owing to its low sensitivity to chemotherapy and radiotherapy, but this results in high mortality, recurrence, and metastasis rates. It is a feasible strategy to construct tumor microenvironments activated by nanotheranostics agents for the diagnosis and therapy of liver cancer. This study reports on a nanotheranostic agent (MONs@PDA-ICG) with manganese oxide nanoflowers (MONs) as core and polydopamine (PDA) as shell loading, with ICG as a photosensitizer and photothermal agent. MONs@PDA-ICG can not only produce ROS to kill cancer cells but also exhibit good photothermal performance for photothermal therapy (PTT). Importantly, O_2_ generated by MONs decomposition can relieve the tumor hypoxia and further enhance the treatment effects of photodynamic therapy (PDT). In addition, the released Mn^2+^ ions make MONs@PDA-ICG serve as tumor microenvironments responsive to MRI contrast for highly sensitive and specific liver cancer diagnosis.

## Introduction

Liver cancer is the second leading cause of death among all kinds of cancer in the world (Llovet et al., 2016). Most patients are diagnosed at an advanced stage, missing a more treatable stage. At present, because liver cancer is not sensitive to chemotherapy and radiotherapy, surgery is the main treatment. However, the recurrence and metastasis rate is 40–70% in the 5 years after operation (Llovet et al., 2005). Therefore, a new treatment with high sensitivity, specificity, and therapeutic efficacy for liver cancer is required.

Photodynamic therapy (PDT) is a promising treatment of cancer that has attracted increasing attention in recent years, owing to its high selectivity, noninvasiveness, low side effects, and no drug resistance (Agostinis et al., 2011; Lan et al., 2019). Indocyanine green (ICG) is a NIR photosensitizer that has been approved by The US food and drug administration (FDA) for use in clinical diagnosis and therapy (Luo et al., 2011). ICG can not only be excited by NIR light to generate reactive oxygen species (ROS, i.e., singlet oxygen, ^1^O_2_) but can also transfer NIR light energy to heat, killing the cancer cells through local high temperature (Trachootham et al., 2009; Yuan et al., 2015; Zhou et al., 2016). The synergistic effect of PDT and photothermal therapy (PTT) can improve the treatment efficacy. However, O_2_ is the key factor of PDT, the hypoxia of tumor microenvironments leads to unsatisfactory treatment effects, severely limiting the further application of PDT in clinical settings (Fan et al., 2015; Chen et al., 2018; Liu et al., 2019; Sahu et al., 2020). Therefore, adjusting the TME is a promising strategy to boost the treatment efficacy of PDT.

Early diagnosis plays an important role in ameliorating the prognosis in patients with liver cancer due to its long incubation period, high mortality, and extremely poor prognosis. Molecular imaging technology is the common diagnostic method for liver cancer, including MRI, CT, ultrasonography, and so on (Janib et al., 2010). However, it is difficult to distinguish small liver cancers from cirrhotic nodules by conventional diagnostic imaging technologies. Tumor microenvironments are closely related to tumor formation and metastasis, featuring low pH, hypoxia, a high concentration of glutathione (GSH), and overexpression of hydrogen peroxide (H_2_O_2_) (Bo et al., 2015; Dai et al., 2017). Exploring the responsive imaging probes of the tumor microenvironments could improve the sensitivity and specificity of liver cancer, as they generate an imaging signal in the tumor site.

**SCHEME 1:**
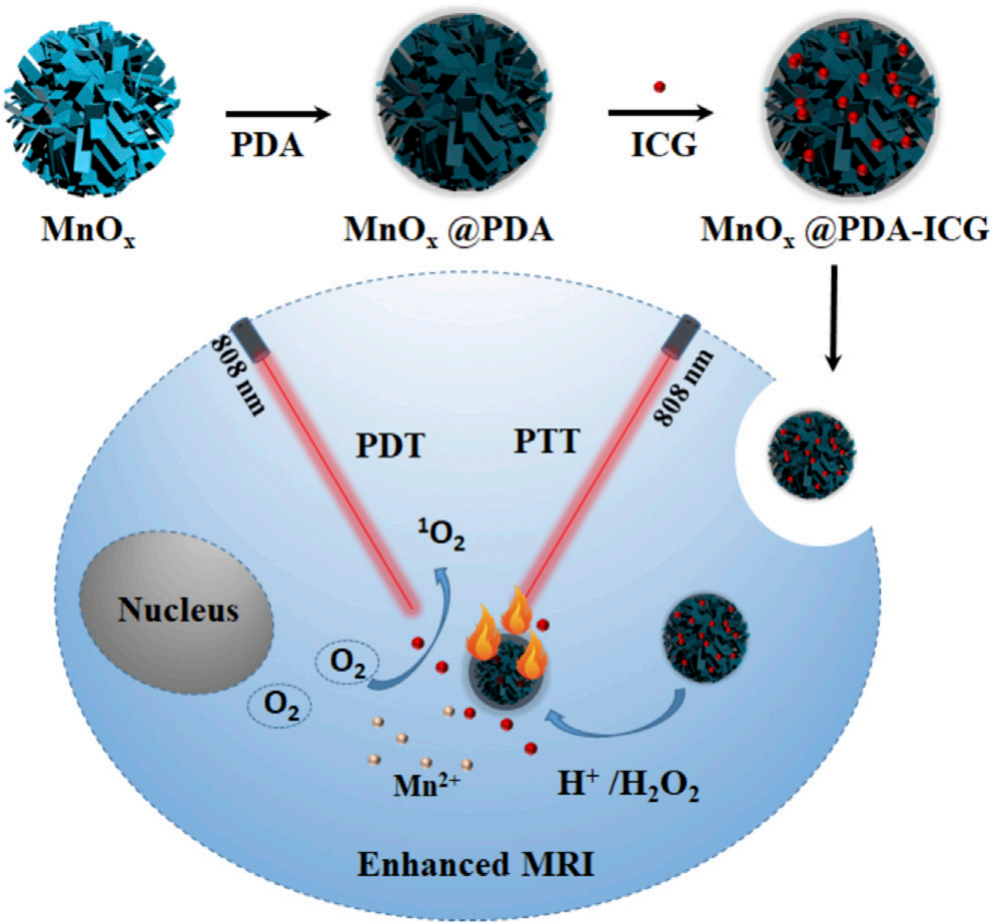
Schematic illustration of MONs@PDA-ICG for enhanced T_1_-guided synergetic photodynamic and photothermal therapy.

The present study constructed a nanotheranostic agent with manganese oxide nanoflowers (MONs) as core and polydopamine (PDA) as shell loading with ICG as a photosensitizer and photothermal agent (MONs@PDA-ICG) for tumor microenvironments responsive MRI and PDT/PTT synergistic therapy of liver cancer (Scheme 1). Under 808 nm laser illumination, MONs@PDA-ICG can not only produce ROS to kill the cancer cells but also exhibits good photothermal performance. More importantly, MONs core in tumor microenvironments (acidic conditions can produce O_2_ to improve the treatment effect of PDT. In addition, the paramagnetic Mn^2+^ ions obtained by MONs core disintegration can achieve pH-activated T_1_-weighted MRI (Kim et al., 2013; Banobre-Lopez et al., 2018; He et al., 2019). Therefore, MONs@PDA-ICG is promising for the diagnosis and treatment of liver cancer with high sensitivity, specificity, and treatment effects.

## Materials and Methods

### Materials

Potassium permanganate (KMnO_4_), Tris (hydroxymethyl) aminomethane (Tris), ethanol, and H_2_O_2_ (30%) were obtained from the Beijing Chemical Reagents Company. Oleic acid (OA, >90%) and dopamine hydrochloride were from Aladdin. 1,3-Diphenylisobenzofuran (DPBF) was separately purchased from Alfa Aesar and Sigma-Aldrich, and CCK-8 was purchased from Changchun Sanbang Pharmaceutical Technology Co (Changchun, China).

### Synthesis of MONs

Manganese oxide nanoflowers were synthesized by a previously reported method (Ding et al., 2020). In brief, 50 ml solutions containing 0.1 g KMnO_4_ were stirred for 30°min, then 1 ml OA was dropped slowly into the mixture and vigorously stirred for another 5 h at room temperature. The dark brown precipitates were dispersed in deionized water after washing with water and ethanol.

### Synthesis of MONs@PDA

10 mg MONs and 24 mg Tris were dissolved into 70 ml deionized water. Then 10 mg dopamine hydrochloride solutions (1 mg ml^−1^) were added drop by drop under ultrasound and stirring. After 2 h, the color of the mixture gradually changed from dark brown to black, and it was then subject to another 8 h stirring. The black solutions were then washed with water and stored at 4°C for future use.

### ICG Loading on MONs@PDA

MONs@PDA-ICG was performed by mixing 5 mg ICG and 5 mg MONs@PDA in 5 ml deionized water and stirring overnight in the dark at room temperature. They were then gently washed twice with deionized water and all the washing supernatants were collected to calculate the ICG loading content by UV−vis.

### Extracellular O_2_ Generation From MONs@PDA

60 μL H_2_O_2_ (1 M) was added into MONs@PDA (150 μg ml^−1^, 30 mL) under stirring. Then the dissolved oxygen meter was used to monitor the generated concentrations of O_2_.

### Extracellular ^1^O_2_ Detection

0.4 mg MONs@PDA-ICG were dispersed in a 2 ml solution containing ethanol and water at a ratio of 6:4. Then 0.5 μl H_2_O_2_ (1 M) and 20 μl DPBF (10 mM) were added. Subsequently, the mixture was exposed to 808 nm irradiation (0.8 W cm^−2^) for different times (0, 2, 4, 6, 8, 10, 15, 20, and 30 min) and the ^1^O_2_ by UV−vis was measured.

### Photothermal Effect and Thermal Stability of MONs@PDA-ICG

Configured solutions with different MONs@PDA-ICG (0, 25, 50, 100 μg ml^−1^) were irradiated with 808 nm laser for 10 min (1.5 W cm^-2^) and measured by thermocouple probe to record the changing temperature every 30 s. Every 2 min, thermal photos were taken by an infrared thermal imaging camera. For the evaluation of thermal Stability, MONs@PDA-ICG solution (50 μg ml^−1^) in quartz cuvette was illuminated for 10 min and naturally cooled down to room temperature. These procedures were duplicated three times.

### Cytotoxicity Assay of MONs@PDA-ICG

Human hepatocellular carcinoma (HCC) cell lines (LM3, HepG2, SNU-387) were used to assess the cytotoxicity of MONs@PDA-ICG. They were all seeded in 96-well plates at 37 °C under a 5% CO_2_ humidified incubator. After 24 h of being cultured, different MONs@PDA-ICG instead of the completed medium were added for another 24 h incubation. The cytotoxicity was then detected by the CCK-8 method. 100 μl CCK-8 medium was added for 4 h and the absorbance (450 nm) was measured by a plate reader.

### *In Vitro* Therapy

For in vitro treatment, expose the LM3, HepG2, SNU-387 cells, incubated with various MONs@PDA-ICG (0, 6.25,12.5, 25, 50 μg ml^−1^) for 24 h, to 808 nm laser for 10 min (1.3 W cm^−2^). Then the same method was manipulated to calculate the cell viability.

### *In Vitro* MRI

The MR images were acquired by measuring different MONs@PDA solutions (Mn content: 0.0325, 0.075, 0.15, 0.3, and 0.6 mM) dispersed with different pH (7.4 and 6.5). The T_1_ relaxation time was taken by the clinical 3.0 T MRI scanner.

## Results and Discussion

**FIGURE 1:**
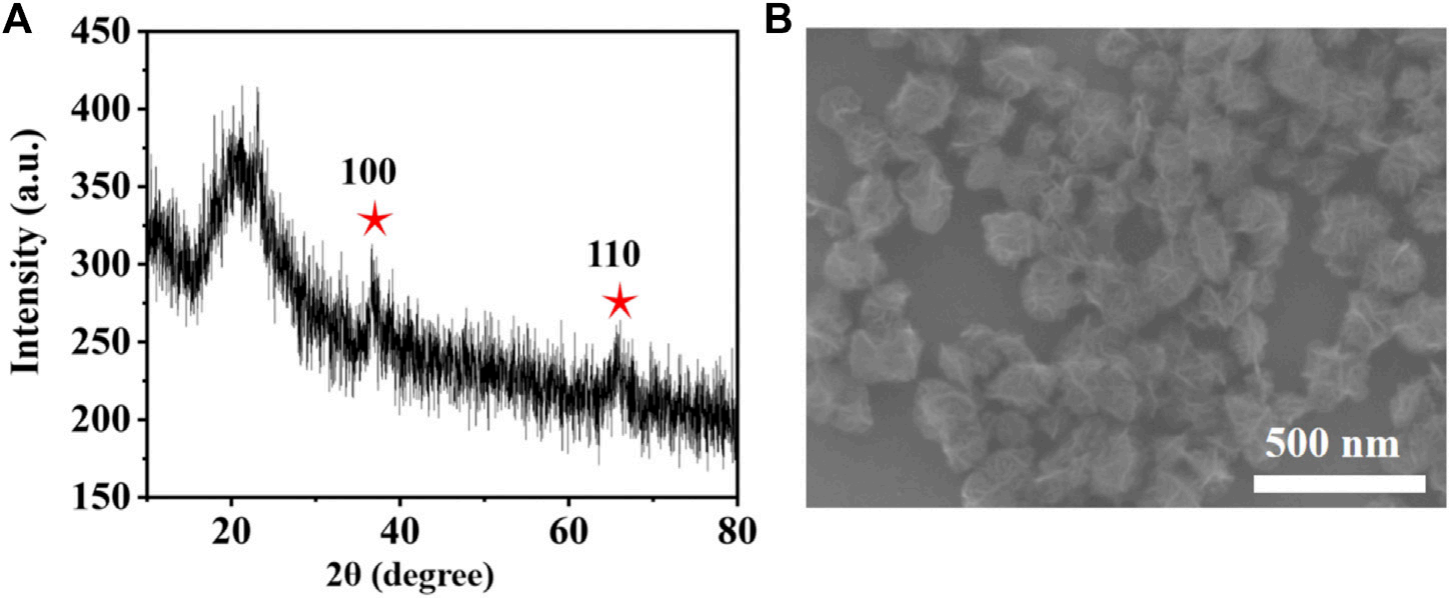
**(A)** XRD patterns of MONs and **(B)** HAADF-STEM images.

**FIGURE 2:**
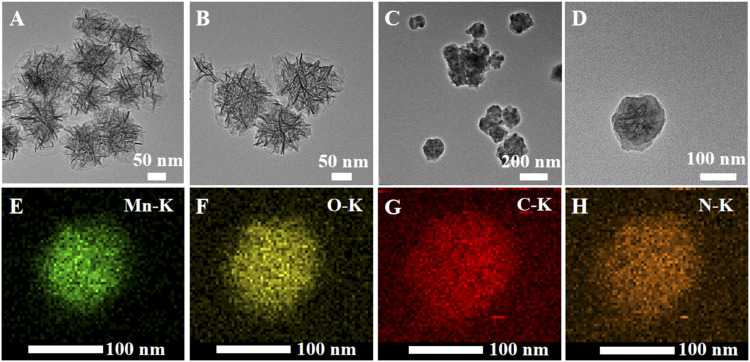
TEM images of **(A,B)** MONs **(C,D)** MONs@PDA **(E–H)** Elemental mapping of Mn, O, C, N.

**FIGURE 3:**
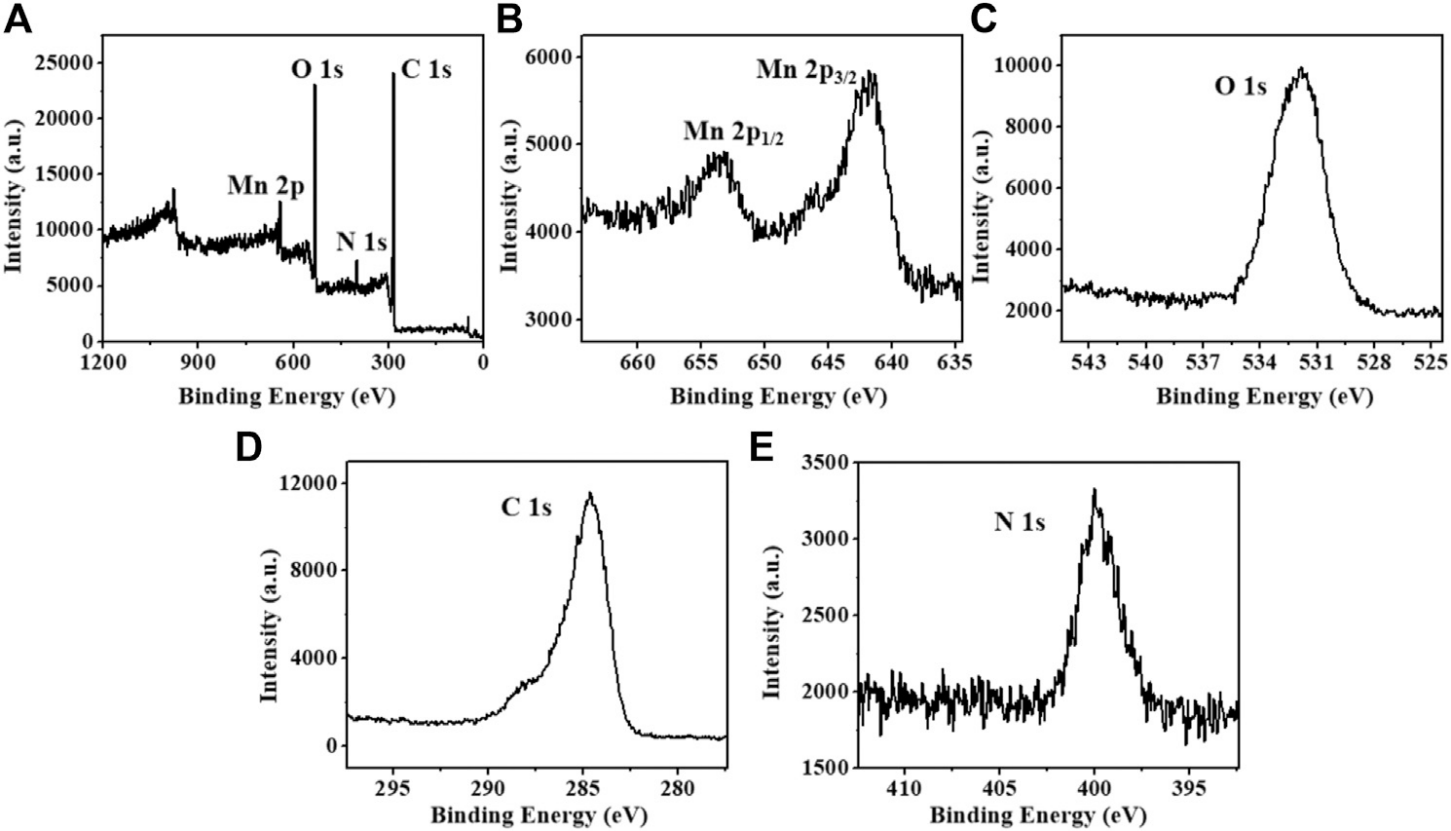
**(A)** XPS spectra of MONs@PDA. **(B-E)** XPS spectra of Mn 2p, O 1s, C 1s and N 1s.

MONs were prepared following previous literature with slight modification (Ding et al., 2020). The X-ray diffraction (XRD) pattern reveals the amorphous structure of MONs (Figure 1A). The scanning electron microscope (SEM) and transmission electron microscope (TEM) images (Figure 1B, Figures 2A,B) show the uniform morphology of MONs with a diameter of about 100 nm. PDA shell was wrapped on the surface of MONs for loading ICG and improving the biocompatibility. As shown in Figures 2C,D, the morphology of MONs@PDA was unchanged after coating with PDA. HAADF-STEM and elemental mapping analysis indicated that Mn, O, N, and C elements were uniformly distributed, demonstrating the successful coating of PDA (Figures 2E–H). The presence of N in the X-ray photoelectron spectra (XPS) of MONs@PDA further confirms that PDA is successfully wrapping (Figure 3). Then, ICG was loading on the surface of MONs@PDA via π–π stacking and hydrogen bonding interactions with a content of 0.372 mg mg^−1^.

**FIGURE 4:**
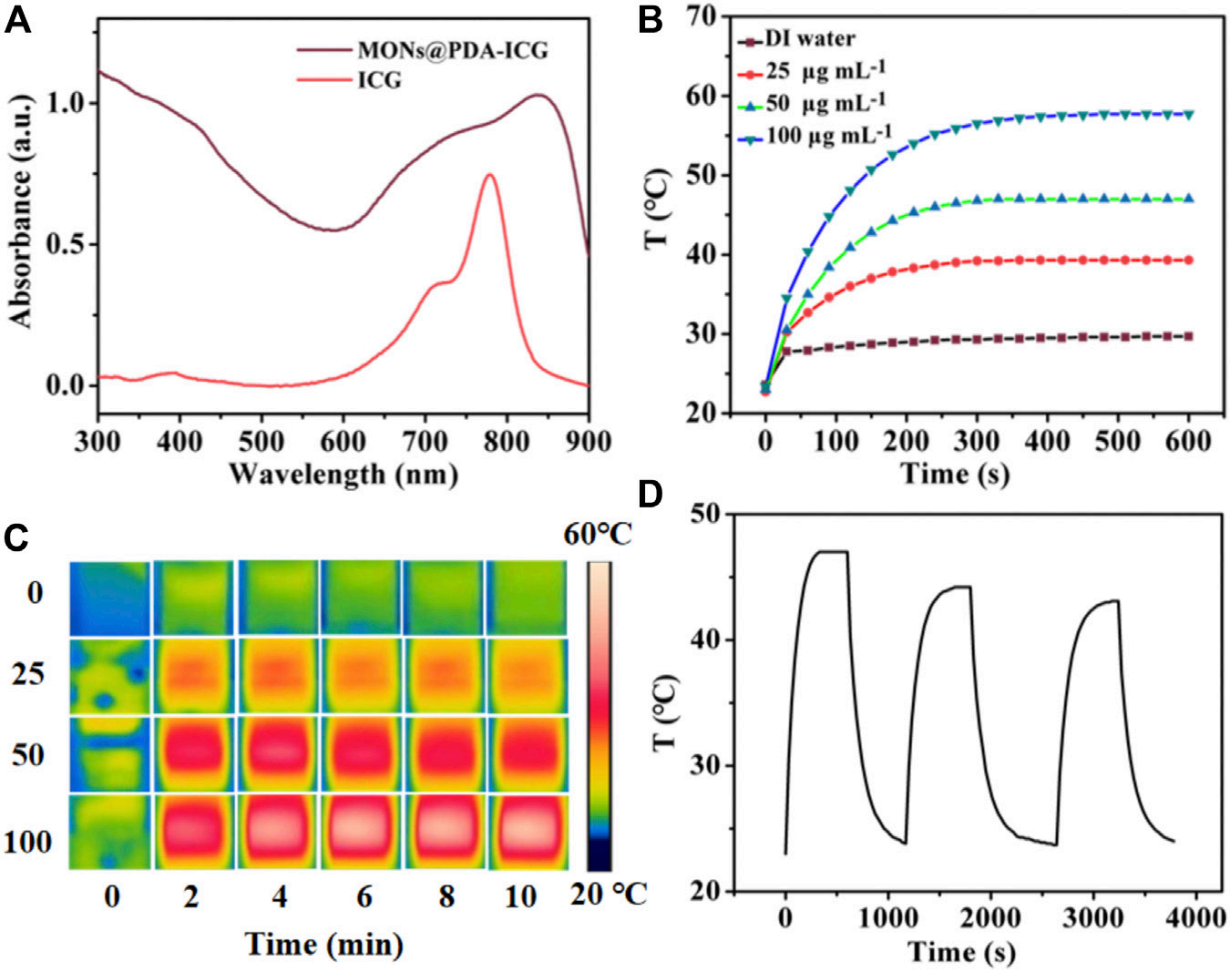
**(A)** UV-vis absorption spectrum of MONs@PDA-ICG and Free ICG. **(B)** Temperature curve of MONs@PDA-ICG with different concentrations under 808 nm laser (1.5 W cm^−2^) for 10 min **(C)** Infrared thermal images every 2 min. **(D)** Cycle curve of MONs@PDA-ICG at 50 μg mL^−1^.

The UV-vis spectrum of MONs@PDA-ICG shows strong NIR absorption owing to the introduction of PDA shell and ICG (Figure 4A). We then investigated the photothermal performance of MONs@PDA-ICG with different concentrations under irradiation with an 808 nm laser (1.5 W cm^−2^). As shown in Figure 4B , compared to the water, the temperature of MONs@PDA-ICG increased from 23.4 °C to 57.7 °C at a concentration of 100 μg ml^−1^ after irradiation 10 min (Figures 4B,C). This high temperature can kill cancer cells (Soares et al., 2012). Alternating heating and cooling experiments show that MONs@PDA-ICG has good photothermal stability after three cycles (Figure 4D) and excellent photothermal conversion efficiency (63.32%). The high photothermal conversion efficiency and good photostability enable MONs@PDA-ICG to act as a promising photothermal agent.

**FIGURE 5:**
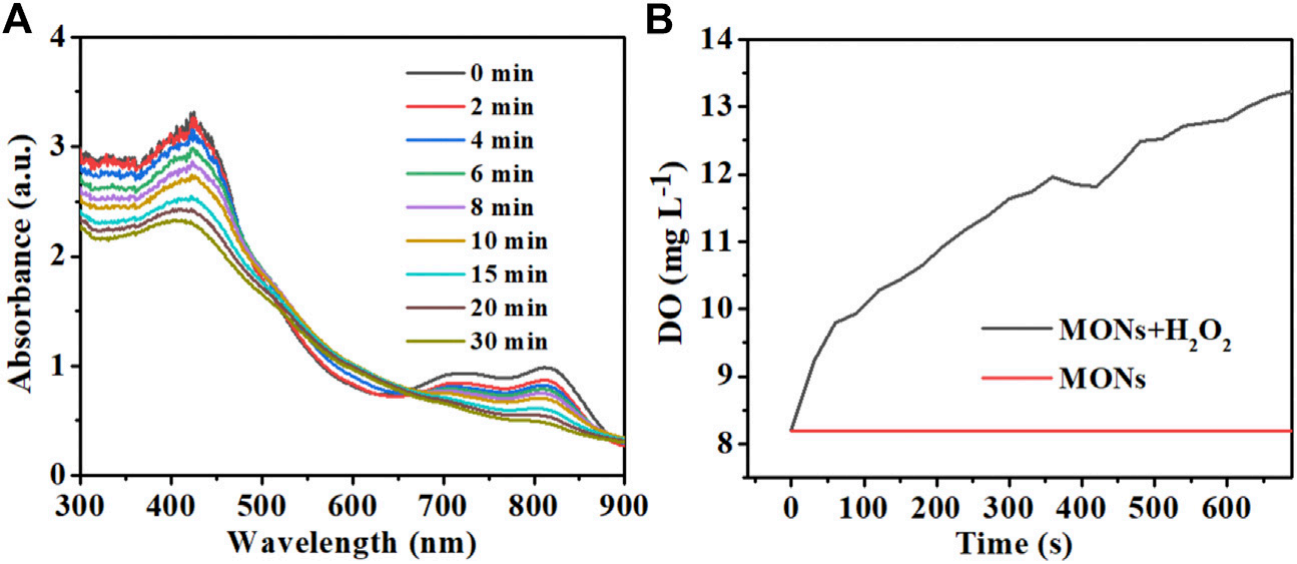
**(A)** UV-vis spectrum of MONs@PDA-ICG with DPBF (20 μL) and H_2_O_2_ (10 μL) at different times for detection of the ^1^O_2_ generation. **(B)** O_2_ generation of MONs@PDA in the presence of 2 mM H_2_O_2_.

ROS generation is the key factor of PDT, so we evaluated the ROS generation ability of MONs@PDA-ICG using the 1,3-diphenylisobenzofuran (DPBF) as the probe whose absorbance at 410 nm decreased in the presence of ^1^O_2_. As shown in Figure 5A, the absorbance of DPBF was diminished with the time of irradiation, extending the 808 nm laser, indicating the production of singlet ^1^O_2_. In addition, the ability of MONs@PDA was investigated in tumor microenvironments to catalyze the H_2_O_2_ decomposition to produce O_2_. We measured the O_2_ generation of MONs@PDA after adding 2 mM H_2_O_2_ in acidic conditions. As shown in Figure 5B, compared with no H_2_O_2_ group, the presence of H_2_O_2_ makes MONs@PDA produce more O_2_ over 10 min. This released that H_2_O_2_ was decomposed and generated O_2_ catalyzed by MONs@PDA, relieving the hypoxia of the tumor. MONs@PDA-ICG is thus promising for highly effective PDT due to this good ROS generation and its ability to modulate tumor microenvironments.

In tumor microenvironments, MONs@PDA can not only catalyze the H_2_O_2_ decomposition to produce O_2_ but also be dissolved into Mn^2+^ ions (Equation 1), achieving a tumor microenvironment responsive MRI (Cai et al., 2019).

$${\text{H}}_{2}{\text{O}}_{2}{+2\text{H}}^{+}{+\text{MnO}}_{\text{X}}{=2\text{H}}_{2}{\text{O}+\text{O}}_{2}{+\text{Mn}}^{2+}$$ (1)

**FIGURE 6:**
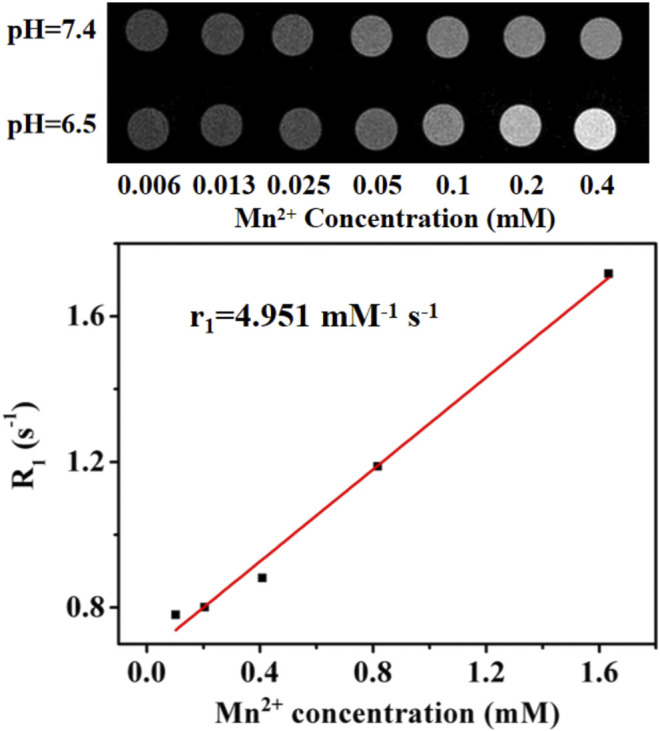
MRI images of MONs@PDA with increased Mn^2+^ content (0.006, 0.013, 0.025, 0.05, 0.1, 0.2, 0.4 mM) at different pH and T_1_ relaxation time.

As shown in Figure 6, the MR signals are unchanged with the increase of MONs@PDA in the normal physiological microenvironment (pH 7.4), but the MR signals became stronger at the same concentration of MONs@PDA tumor microenvironment (pH 6.5 and 2 mM H_2_O_2_), with the longitudinal relaxation (r_1_) value of 4.951 mM^−1^ s^−1^. The results show that MONs@PDA could act as a tumor microenvironment responsive MRI contrast for highly sensitive and specific liver cancer diagnosis.

**FIGURE 7:**
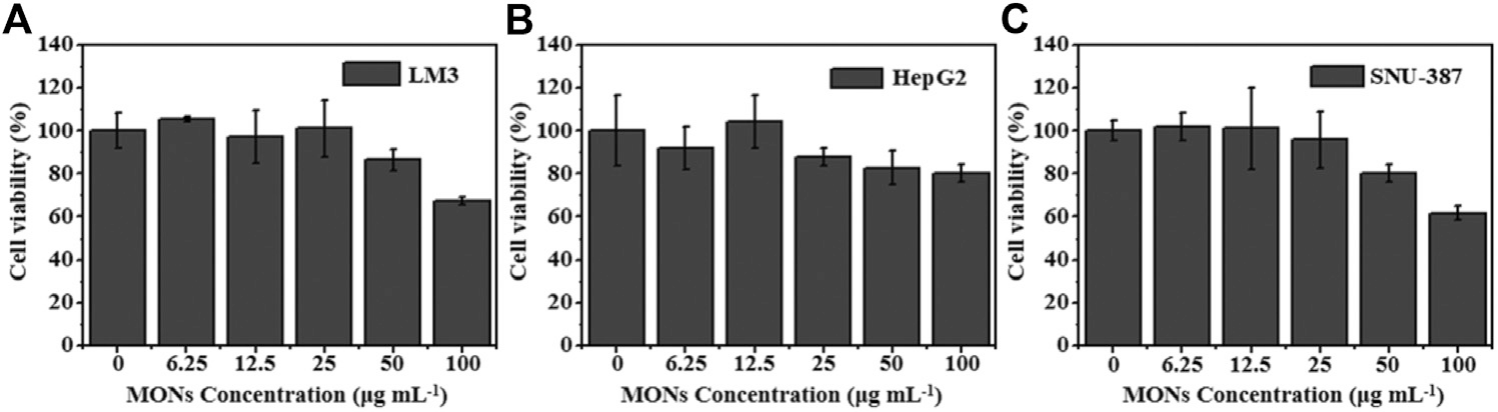
The cell viability of **(A)** LM3, **(B)** HepG2 and **(C)** SNU-387.

**FIGURE 8:**
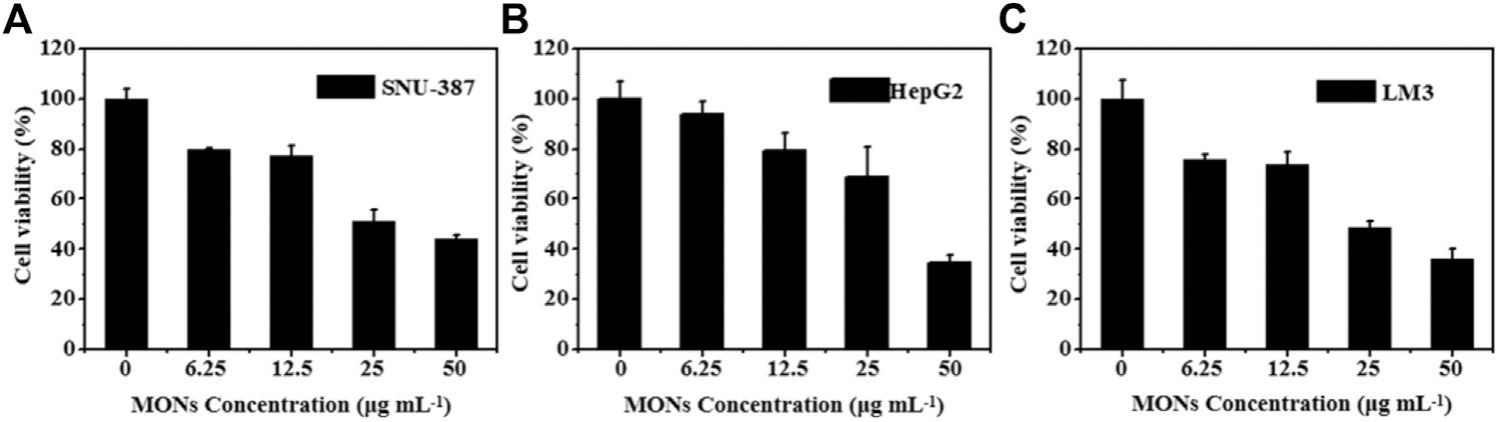
The therapy effect *in vitro*. **(A)** SNU-387 **(B)** HepG2, and **(C)** LM3 were exposed to 808 nm irradiation for 10 min (1.3 W cm^−2^).

Encouraged by the good ROS generation capacity and photothermal properties, we further investigated the potential of MONs@PDA-ICG as a nanotheranostic agent for synergistic PTT and PDT *in vitro*. The LM3, HepG2, SNU-387 were chosen to assess the cytotoxicity of MONs@PDA by cell counting kit 8 (CCK-8) assay. As shown in Figure 7, after incubating with MONs@PDA with different concentrations (6.25–100 μg/ml) for 24 h, the cell viabilities were higher than 80% at 50 μg ml^−1^ for LM3, HepG2, and SNU-387, indicating their good biocompatibility. Then, the treatment effect of synergistic PTT and PDT was evaluated using LM3, HepG2, SNU-387. After irradiation with 808 nm NIR laser for 10 min, the cell viability of LM3, HepG2, SNU-387 cells was no more than 40% (Figure 8), displaying the highly efficient therapeutic efficacy of synergistic PTT and PDT for liver cancer.

## Conclusion

In summary, we successfully synthesized a nanotheranostic agent for tumor microenvironment responsive MRI and combinatorial PTT and PDT for liver cancer. The designed MONs@PDA-ICG worked as both a photosensitizer and photothermal agent under 808 nm NIR irradiation. Meanwhile, MONs@PDA-ICG can release oxygen and Mn^2+^ ions in tumor microenvironments. The oxygen produced, relieved the tumor hypoxia and further enhanced the treatment effect of PDT. In addition, the release of Mn^2+^ ions makes MONs@PDA-ICG serve as a tumor microenvironment responsive MRI contrast for highly sensitive and specific liver cancer diagnosis. This outstanding treatment effect was demonstrated by the representative liver cancer cells, including LM3, HepG2, SNU-387. These findings validate the conclusion that the nanotheranostic agent based on MONs@PDA has potential in future liver cancer diagnosis and therapy.

## Data Availability

The original contributions presented in the study are included in the article/Supplementary Material, further inquiries can be directed to the corresponding authors.
